# Global DNA-methylation in quantitative epigenetics: orbitrap mass spectrometry

**DOI:** 10.3389/fmolb.2025.1681568

**Published:** 2025-09-26

**Authors:** Janine F. M. Otto, Georg Pohnert, Thomas Wichard, Michael Bauer, Anne Busch, Nico Ueberschaar

**Affiliations:** ^1^ Department of Instrumental Analytics/Bioorganic Analytics, Institute for Inorganic and Analytical Chemistry, Friedrich Schiller University Jena, Jena, Germany; ^2^ Cluster of Excellence, Balance of the Microverse, Friedrich Schiller University Jena, Jena, Germany; ^3^ Chemical Ecology of Cross Kingdom Interactions, Institute for Inorganic and Analytical Chemistry, Friedrich Schiller University Jena, Jena, Germany; ^4^ Department of Anaesthesiology and Intensive Care Medicine, Jena University Hospital, Jena, Germany; ^5^ Department of Theoretical Microbial Ecology, Institute of Microbiology, Friedrich Schiller University Jena, Jena, Germany; ^6^ Mass Spectrometry Platform, Friedrich Schiller University Jena, Jena, Germany

**Keywords:** global DNA methylation, 5-methylcytosine, epigenetics, *Ulva mutabilis*, UHPLC-HRMS, high-throughput

## Abstract

DNA methylation is the most common epigenetic modification in both prokaryotic and eukaryotic genomes. Here we present a method based on highly efficient acid-hydrolysis of DNA, liquid chromatography, and detection by mass spectrometry to accurately quantify cytosine methylation in highly methylated DNA samples. This approach enables direct, rapid, cost-efficient, and sensitive quantification of the methyl-modified nucleobase 5-methylcytosine and 6-methyl adenine, along with their unmodified nucleobases. In contrast to standard sequencing techniques, our method only gives quantitative information on the overall degree of methylation, but it requires only small amounts of DNA and is not dependent on lengthy bioinformatic analyses. Our method allows rapid, global methylome analysis and quantifies a central epigenetic marker. In a proof-of-principle study, we show that it can also be extended to the monitoring of other DNA modifications, such as methylated adenine. Uncomplicated data analysis facilitates a quick and straightforward comparison of DNA methylation across biological contexts. In a case study, we also successfully identified changes in methylation signatures in the marine macroalga *Ulva mutabilis* “slender”. The advantage of global methylation analysis compared to sequencing allows for generating fast prior knowledge on which sample sequencing is senseful. The great benefit of the presented method is the speed and accuracy of the global methylation analysis, which is independent of the total methylation rate and gives accurate results, whereas competitive based on enzymatic digestion might fail.

## Graphical Abstract

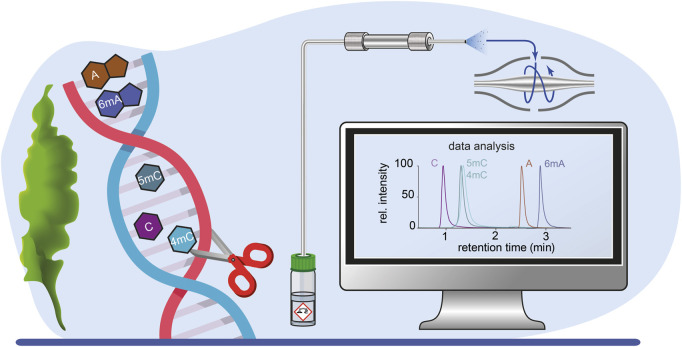

## 1 Introduction

DNA encodes the genetic information in all domains of life and is the central molecule in the heredity of species-specific traits. Next to the four nucleobases adenine, cytosine, thymine and guanine, eukaryotic DNA predominantly contains 5-methylcytosine (5 mC) as a modified nucleobase. DNA methylation and related modifications have distinct functions in cells, such as regulating gene expression, maintaining the genome, controlling DNA replication, and, for eukaryotes, organizing chromatin structure (Casadesus and Low, 2006; Wion and Casadesus, 2006; Gouil and Keniry, 2019). Investigation of DNA methylation is a key element in the field of epigenetics, which studies how cells control gene activity without changing the DNA sequence (Waddington, 2012).

The detection of methylated sites is commonly enabled by methylation-sensitive sequencing techniques. Short read sequencing combined with bisulfite sequencing can detect 5 mC in genomic DNA (Krueger et al., 2012; Adusumalli et al., 2014; Searle et al., 2023) and provides information about the location of 5 mC in a genomic context. Nevertheless, there are limitations due to harsh conditions and the misidentification of 4 mC (Tanaka and Okamoto, 2007). Long-read sequencing methods, such as PacBio Single Molecule Real-Time sequencing (SMRT) and Oxford Nanopore sequencing (ONT), can indirectly detect multiple forms of DNA modifications (Barros-Silva et al., 2018; Hu et al., 2021; Searle et al., 2023). SMRT and ONT technologies enable the differentiation between modifications as 6mA, 5mC, and 4 mC in a genomic context, but this relies on known methylation sites within palindromes found in databases like REBASE (Roberts et al., 2015), meaning unknown methylations and other unusual modifications may be challenging to unambiguously identify their chemical structure(Beaulaurier et al., 2019; Gouil and Keniry, 2019). New developments in nanopore based sequencing and *de novo* detection of modification allows to track sequencing information on potential new DNA modifications (Liu et al., 2024). Nevertheless, these techniques are expensive, time-consuming, and require complex bioinformatic data analysis (Gouil and Keniry, 2019). Furthermore, the amplification-free protocols for long-read sequencing require high amounts of high-quality DNA. Therefore, it is challenging to investigate the epigenetics of non-model organisms or complex communities for which no cultivation methods or reference genomes are available.

On the other hand, analyses based on high-performance liquid chromatography coupled to mass spectrometry (HPLC-MS) of hydrolyzed DNA nucleosides or nucleobases, in principle, allow the detection of any DNA modification and absolute quantification independent of the sequence context (Gao et al., 2019; Traube et al., 2019; Varma et al., 2022; Adamczyk et al., 2023). An analysis of the proportion of methylated and unmethylated nucleobases (global methylome analysis), comparing at least two system states, is often sufficient to describe the epigenetic regulation of processes. The advent of rapid and cost-effective global methylation methods has engendered a paradigm shift in the field, enabling the comparison of a multitude of samples. This advancement has emerged as a significant advantage over conventional sequencing methods. It is an established fact that, in general, global methylation methods generate prior knowledge on which sample a sequencing is senseful. Several analytical approaches have been developed for the analysis of global DNA methylome, with mass spectrometry enabling sensitive and precise analysis of small molecules. In contrast, DNA molecules cannot be easily ionized, and since mass spectrometry is more sensitive for nucleosides compared to nucleotides, additional dephosphorylation is required (Chen et al., 2024). This includes matrix assisted laser desorption and ionization mass spectrometry (MALDI-MS) (Humeny et al., 2003; Xie et al., 2024), gas chromatography-mass spectrometry(GC-MS) (Romerio et al., 2005), and LC-MS-based methods (Schiffers et al., 2017; Traube et al., 2019; Zhang et al., 2021; Chen et al., 2024).

A challenge is the quantitative hydrolysis of DNA into single analyzable nucleosides or nucleobases without destroying the methylation patterns. Most approaches use enzymatic hydrolysis of DNA into nucleosides. However, enzymes are constrained in their hydrolysis efficiency in cases of high covalent DNA modification rate. Further, matrix effects, which lead to incomplete digestion of genomic DNA or an unavoidable nucleoside background caused by the enzymes themselves, are encountered (Park et al., 2003; Dong et al., 2012; Tretyakova et al., 2013; Schiffers et al., 2017; Lowenthal et al., 2019).

Only a few reports on chemical hydrolysis are available, but to our knowledge, they have not been thoroughly validated (Kok et al., 2007; Yamagata et al., 2012; Ueberschaar et al., 2013; Vető et al., 2018; Lowenthal et al., 2019). The commonly used formic acid treatment leads to formylated side-products that prevent quantitative analysis. Here, we established and applied an HCl-based hydrolysis protocol that releases methylated and unmethylated nucleobases, which can be directly submitted to a high-throughput ultra-high-performance liquid chromatography coupled with high-resolution mass spectrometry (UHPLC-HRMS).

As proof of concept, we investigated the DNA methylation levels of the green macroalga and model organism *Ulva mutabilis* (*U. mutabilis*) under standardized culture conditions (Wichard, 2023), aiming to understand methylation dynamics in the presence or absence of co-occurring bacterial symbionts that release growth- and morphogenesis-promoting factors (AGMPFs).

*U. mutabilis* (Chlorophyta), also known as sea lettuce, is one of the most abundant green seaweed species in coastal benthic environments worldwide and is capable of forming extensive green tides (Smetacek and Zingone, 2013). It is characterized by a high level of global DNA methylation, attributed to its densely methylated CpG content (Pakhomova et al., 1968; Gupta et al., 2012; Gupta et al., 2015; Oertel et al., 2015).

**FIGURE 1:**
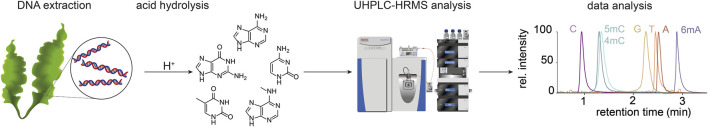
Analysis scheme for the detection of global DNA modifications using acid hydrolysis followed by UHPLC-HRMS analysis.

Our findings highlight the advantages of chemical (acidic) hydrolysis as a robust and broadly applicable method for analyzing highly methylated *U. mutabilis* DNA, offering improved efficiency over conventional enzymatic approaches (see Figure 1).

## 2 Materials and methods

### 2.1 Reagents

Chemicals were purchased from the following suppliers: cytosine, 5-methylcytosine, 2ˈ-deoxycytidine, and uracil were purchased from Sigma-Aldrich GmbH, Taufkirchen, Germany; 2ˈ-deoxy-5-methylcytidine from VWR, Avantor, Darmstadt, Germany, 2ˈ-deoxy-*N*-4-methylcytidine from Biosynth Ltd., Compton, UK, 2ˈ-deoxycytidine-^13^C_1_, ^15^N_2_ (C-^13^C_1_^15^N_2_) and 2ˈ-deoxy-5-methylcytidine-^13^C_1_, ^15^N_2_ (5mC-^13^C_1_^15^N_2_) from Toronto Research Chemicals Inc., North York, Canada. All compounds were dissolved at 1 mg/mL in water for further dilution if necessary. DNA standards with either 100% unmodified or methylated cytosines were purchased from Zymo Research Europe GmbH, Freiburg, Germany.

### 2.2 Biological resources

*Ulva mutabilis* Føyn morphotype ‘slender’ (strain FSU-UM5-1) was cultivated in *Ulva* Culture Medium (UCM) (Stratmann et al., 1996; Califano and Wichard, 2018) only in the presence of the bacteria *Roseovarius* sp. MS2 and *Maribacter* sp. MS6, forming a tripartite community, or under axenic conditions (absence of any bacteria in the culture) for 2 weeks (Califano and Wichard, 2018). Cultures were maintained at 18 ± 2 C, with a 17-h light/7-h dark period, and light intensity was kept between 40 and 80 µmol photons m^−2^s^−1^. *U*. *mutabilis* was cultured under strictly standardized conditions as a haploid strain propagating asexually, yielding synchronized clonal populations with minimal variance among biological replicates. Therefore, technical triplicates of three biological clonal specimens were used for analysis. Notably, *U. mutabilis* was recently reclassified as *Ulva compressa*; however, we retain the cultivar name *U. mutabilis* for consistency with the literature (Steinhagen et al., 2019).

### 2.3 DNA extraction and analysis

*U. mutabilis* genomic DNA was extracted from freeze-dried and homogenized algal tissue with the Qiagen DNeasy® Plant Mini Kit. 50 μg RNase I was added during the cell lysis step. (RNA-free preparation was confirmed by monitoring of uracil, which was not detected).

### 2.4 DNA acid hydrolysis

One µg DNA standard (Zymo Research Europe GmbH, Freiburg, Germany) was diluted to 20 ng with water and used for method development. The extracted DNA from *U*. *mutabilis* was eluted from the DNA extraction column with water with a minimum concentration of 2 ng/μL. (Note: elution with buffer resulted in an inadequate quantification possibility due to a decreased response and bad peak shape). All used DNA or nucleosides were transferred into glass vials (WICOM Germany GmbH, Heppenheim, Germany, 1.5 mL, 8 mm) with inserts (VWR International GmbH, Darmstadt, Germany, micro-insert 0.1 mL, 15 mm top). Ten µL of appropriate amounts of internal standard (IS) (200 nM C-^13^C_1_^15^N_2_ and 44 nM 5mC-^13^C_1_^15^N_2_, diluted 1:4 during hydrolysis) and 10 µL of 8% HCl (2% final concentration) were added, and the reaction mixture was filled up to 40 µL with water. Vials were sealed airtight with a Teflon™-coated silicone cap, and samples were heated to 120 C for 3 h (NOTE: wide bore vials with 9 mm caps proved to be not sufficiently tight and heat resistant for a successful hydrolysis). After cooling to room temperature, 100 µL H_2_O was added to dilute the reaction mixture and enable a freezeable concentration for the following freeze-drying. Afterwards, hydrolyzed DNA fragments were re-dissolved in 40 µL H_2_O by vortexing the vials and analyzed by an UHPLC-HRMS. For the generation of the calibration curves, deoxycytidine and 5-methyl deoxycytidine were dissolved in H_2_O and diluted to give stock solutions of 800 nM. These were combined and diluted to the appropriate concentrations. To these solutions, stable isotope-labeled standards were given (final concentration of 50 nM). These solutions were acid-hydrolyzed as described above.

### 2.5 DNA enzymatic digestion

Enzymatic DNA hydrolysis was performed using DNA Degradase Plus™ (Zymo Research Europe GmbH, Freiburg, Germany). 20 ng DNA standard in modified TE buffer (10 mM Tris-HCl; 0.1 mM ETDA; pH 8.0) (Zymo Research Europe GmbH, Freiburg, Germany) was mixed with 1 µL DNA Degradase Plus™ (5 units/µL) and 4 µL 10× DNA Degradase™ Reaction Buffer and kept at 37 C for 1–4 h. Then 10 µL 200 nM IS were added and filled up to 40 µL with H_2_O. Afterwards, samples were filtered through 4 mm Millex®-GV hydrophilic PVDF syringe filters (0.22 µm, Merck, Sigma-Aldrich Chemie GmbH, Taufkirchen, Germany). For a combination of enzymatic and chemical hydrolysis, DNA standards were first digested with the nucleases and afterwards treated with acid as described above.

### 2.6 UHPLC-HRMS analysis

Analysis of all samples was performed with a UHPLC-HRMS. For UHPLC, an UltiMate 3000 UHPLC system (Thermo Fisher Scientific, Bremen, Germany) with HPG-3400 rapid separation binary pump, TCC-3200 column department, and WPS-3000 autosampler was used. One to 2 µL of samples were injected by an autosampler set to 10 °C and equipped with a 25 µL injection syringe and a 100 µL sample loop. For evaluation three different HPLC columns (Thermo™ Accucore C-18 RP (100 × 2.1 mm, 2.6 µm), Phenomenex Synergi™ Hydro-RP (150 × 2.00 mm, 4 µm), and Phenomenex Synergi™ Fusion-RP (100 × 2 mm, 2.5 µm) were tested with different solvents (A_1_: H_2_O+ 2% acetonitrile +0.1% FA, A_2_: 20 mM HCOONH_4_, pH 2.8, A_3_: 20 mM HCOONH_4_, pH 4.3; B: acetonitrile). The following gradient was applied with a constant flow rate of 0.4 mL/min after method optimization: 100% A_1-3_ for 0.2 min, linear gradient to 50% B in 4 min, column rinsing for 2 min with 100% B, and re-equilibration with 100% A_1-3_ for 1 min.

Mass spectra were recorded with a Thermo Scientific Q Exactive plus™ hybrid quadrupole-Orbitrap mass spectrometer (Thermo Fisher Scientific, Bremen, Germany) equipped with a heated electrospray source (HESI). To monitor the full scan in positive mode, the following parameters were selected: scan range: 80–800 m/*z*, resolution: 35,000, Automated Gain Control (AGC) target: 5 × 10^5^; maximum injection time (IT): 64 m. For MS^2^ experiments in parallel reaction monitoring (PRM) following settings were applied: full MS resolution: 35,000, PRM resolution: 17,500, AGC target 1 × 10^5^, maximum IT: 100 m, isolation window: 1.0 m/*z*, fixed first mass: 50 m/*z*, normalized collision energy (NCE): 150 for a precursor ion with *m*/*z *126.0662. General settings: sheath gas flow rate: 60; auxiliary gas flow rate 20; sweep gas flow rate: 6; spray voltage: 3.0 kV; capillary temperature: 360 C; S-lens radio frequency (RF) level: 50; auxiliary gas heater temperature: 400 C; acquisition time frame: 0.4–7 min.

### 2.7 Data analysis

MS-Data analysis was performed using Thermo Scientific™ FreeStyle™ Version 1.8SP2 and Xcalibur™ Version 4.5.445.18 Quan Browser software (Thermo Fisher Scientific, Bremen, Germany). For peak detection and quantification of the nucleobases, the following settings were applied: retention time window = 20 s; signal = XIC from full scan; peak detection algorithm = ICIS (Smoothing = 9); peak detection mode = highest peak; mass tolerance = ±8.0 ppm. Samples were measured as triplicate if not stated otherwise.

The following equation was used to calculate the relative number of methylated nucleobases in percentage (c is the respective concentration):

$$\frac{c\left(5mC\right)}{c\left(5mC\right)+c\left(C\right)}\times 100$$

Limit of detection and limit of quantification LOD/LOQ values in percentage of the total amount of unmodified nucleobases were calculated using the upper LOQ value of the unmodified base and the lower LOD/LOQ value of the modified base in the given equation.

### 2.8 Statistical analysis

External calibration curves with 11 calibration standards in triplicate (intra-day replication) were made to quantify cytosine and 5-methylcytosine, as well as 4-methylcytosine, adenine, and 6-methyladenine. No weighting was applied here. The linear regression model was calculated, plotted, and analyzed with Origin 2023. Parameters for the calibration (LOD and LOQ) were determined according to DIN 32645 (for mathematical formulas, see Supplementary Material) (Reichenbächer and Einax, 2011). For the statistical values of accuracy and precision, see Supplementary Tables S2–S15).

## 3 Results

To develop and apply a new method for the quantification of (methylated) cytosine using aqueous acidic conditions, several aspects of the analytical process need to be established and verified. This includes the hydrolysis conditions to release all the nucleobases without destroying them using too harsh conditions. Second, an appropriate analytical method for separation and detection has to be established, including calibrations. As the most important fact, we compared our method to known enzymatic-based methods to show the benefits of our new method for our high-methylated green macroalgal system.

### 3.1 Hydrolysis optimization

**FIGURE 2:**
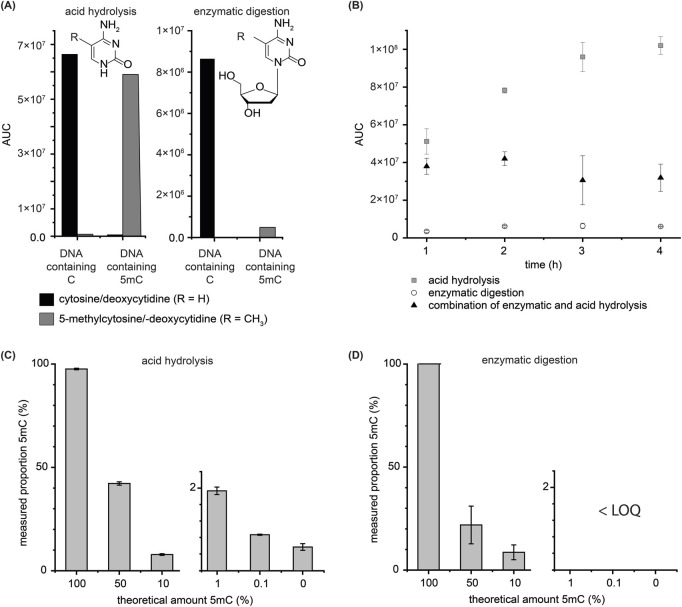
Comparison of enzymatic hydrolysis activity with acidic hydrolysis of (per-methylated) synthetic DNA. **(A)**Area under the curve (AUC) values of free nucleobases after acid hydrolysis or nucleosides after nuclease digestion of two DNA standards that only contain unmodified cytosine or five methylcytosine. **(B)**AUC values of 5-deoxymethylcytidine after enzymatic digestion and five methylcytosine after a combination of enzymatic and acid hydrolysis and after acid hydrolysis. **(C)**Measured proportion of 5 mC in different mixtures of DNA only containing unmodified or methylated cytosine after acid hydrolysis and **(D)**enzymatic digestion. (Note: for lower concentrations than 10% no 5 mC at all could be detected) All errors result from the standard deviation of the mean value (*n*= 3).

For method development, the acid hydrolysis of DNA to nucleobases was established before optimizing the further steps (Figure 1). Hydrolysis of partially modified DNA gives methylated and unmodified free nucleobases. In contrast, enzymatic DNA cleavage results in free nucleosides and requires other protocols for downstream analysis. To optimize the DNA hydrolysis three acids, namely, formic-, trifluoroacetic- and hydrochloric acid were tested and compared. During hydrolysis with formic acid (Shibayama et al., 2016; Lowenthal et al., 2019) a formyl cytidine adduct was formed. This prevents quantitative analysis since 5-formylcytosine is also a known product from enzymatic DNA modification (see Supplementary Figure S1 for details). We therefore evaluated hydrolysis with aqueous hydrochloric acid (HCl), which can be easily evaporated and is not redox active. Different temperatures, acid concentrations, and hydrolysis times were tested for nucleoside and DNA hydrolysis (see Supplementary Figures S2,3). Quantitative hydrolysis to free nucleobases was achieved after the treatment of DNA with 2% HCl for 3 h at 120 °C. Under these conditions, the hydrolysis products are stable, and methylation is preserved (Figure 2B; Supplementary Figure S4). Reactions were executed in closed standard HPLC-vials with spring and insert heated in a standard aluminum block as a micro-autoclave. Trifluoroacetic acid was also capable of hydrolyzing the nucleosides (Lowenthal et al., 2019). Still, it was less effective compared to HCl (comparable peak area ratios were only reached after longer hydrolysis time). Furthermore, HCl was preferred because it has a better environmental footprint compared to the fluorinated acid.

### 3.2 LC-MS method development

To develop a rapid method that allows baseline separation of the nucleobases with peaks off from column dead volume (0.69 min), we evaluated three different polar modified reversed-phase C18 columns using different buffered aqueous phases and optimized the gradient to achieve separation of cytosine and 5-methylcytosine, within 3 minutes (see Supplementary Material Supplementary Figures S5,6). Best separation was achieved by using the Phenomenex Synergi™ Fusion-RP column (Phenomenex, Torrance, CA, US), a polar-embedded C18 phase with polar and hydrophobic selectivity, and a gradient from 20 mM HCOONH_4_ aqueous solution buffered at pH 4.3 to pure acetonitrile (see UHPLC-HRMS analysis and Supplementary Material Supplementary Figure S6). MS detection was optimized by tuning the AGC target. (NOTE: the hydrolysis method and LC-MS analytics are also able to release and detect other relevant DNA and RNA building blocks like adenine (A), 6-methyladenine (6 mA), guanine (G), thymine (T), uracil (U), and 4-*N*-methylcytosine see Figure 1 and Supplementary Figure S5).

### 3.3 Comparison of enzymatic and acidic hydrolysis

To compare the efficiency of acid hydrolysis with that of enzymatic digestion by DNA Degradase Plus™, DNA standards that either contain only unmodified or only methylated cytosine were cleaved using both protocols. Figure 2A shows clear differences in the integrated peaks (area under the curve, AUC) of free nucleobases after acid hydrolysis and free nucleosides after enzymatic digestion. The absolute differences in the integrals can be explained by the different ionization capabilities of the individual compounds and matrix suppression in enzymatic hydrolysis. The low AUC value of 5 mC detected after enzymatic hydrolysis of the DNA standard that should only contain 5 mC and no C can be explained by a hindrance of enzymatic hydrolysis due to a high degree of methylation. In contrast, acid hydrolysis gives similar AUCs from non-methylated and methylated DNA. The proportion of detected 5 mC and C reflects the composition of the standard. Additionally, we evaluated the hydrolysis using a combination of both methods depending on the reaction time (see Figure 2B). The highest AUC values of 5-methylcytosine were detected using acid hydrolysis only. Similar results were observed for cytosine (Supplementary Figure S7).

We then hydrolyzed different mixtures of the DNAs with cytosine and 5-methylcytosine only with hydrochloric acid or DNA Degradase Plus™ (see Figures 2C,D). The results after acid hydrolysis are far more precise and accurate compared to the results after enzymatic digestion (see Supplementary Table S1). The highest error was detected in the enzymatically hydrolyzed sample containing a 1:1 mixture of fully methylated and unmethylated DNA, where only 21.9 ± 9.2% mC was detected (for comparison: after acid hydrolysis 42.2 ± 0.8%, *n* = 3). In the mixtures with a proportion of 1% 5 mC and lower, digested with the enzymes, no 5 mC could be detected anymore, while clear signals were observed after HCl treatment. (The ratios here, which are above expectations, might be a contamination from the synthetic DNA that was already detected in Figure 2A.) Thus, acid hydrolysis could be used as a universal and accurate method for the global methylome analysis of DNA with high as well as low methylation grades.

### 3.4 Calibration

**FIGURE 3:**
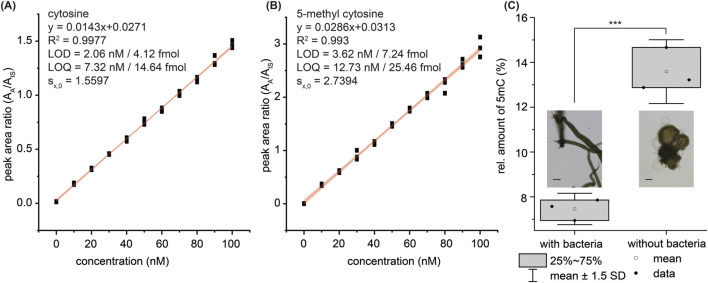
Linear calibration (red line, *n* = 3) of **(A)** cytosine and **(B)** 5-methylcytosine from 0 to 100 nM with 50 nM internal standard for every analyte. The light red area around the linear calibration line displays the confidence interval. LOD = limit of detection; LOQ = limit of quantification; R^2^ = coefficient of determination; s_x,0_ = standard error of the mean. LOD and LOQ are given as concentration (nM, in 2 µL injection volume) and as amount on column (fmol). **(C)** Application of the method in a biological context. The relative level of 5 mC in the eukaryotic green seaweed *Ulva mutabilis* changes significantly depending on the presence or absence of its associated bacteria. *n* = 3 (triplicate of biological clonal specimens; technical triplicate). The images display propagules of *Ulva mutabilis* (left inset bar = 500 µm) and a callus under axenic conditions (right inset, bar = 100 µm). 5mC/total **(C)** 0.75% (LOD), 2.63% (LOQ). Ratios were calculated as described in the Experimental Section. The error bars result from the standard deviation of the mean value. Student’s t-test: p < 0.001 - ***.

A calibration using stable isotope-labeled internal standards was performed to quantify the nucleobases C and 5 mC. For that, the respective nucleosides were hydrolyzed with the optimized method. Several calibration ranges were applied to cover large differences in the concentration of nucleobases that might occur in different organisms. The calibration function from 0 to 100 nM, the respective R^2^-value, and standard error of the mean (s_x,0_) are given in Figures 3A,B. Further calibration functions with extended calibration ranges are given in the Supplementary Material Supplementary Figures S8,9. The limit of detection (LOD) and limit of quantification (LOQ) are in the low picomolar to low femtomolar range, which makes this method perfectly suitable for the detection of low amounts of methylated nucleobases and low quantities of necessary DNA. This LOD/LOQ is comparable to reported enzymatic methods (Varma et al., 2022).

### 3.5 Quantification of additional nucleobases

In addition to the quantification of 5-methylcytosine (5 mC) and cytosine (C), the method allows the reliable detection of other methylated nucleobases like 4-*N*-methylcytosine (4 mC), adenine (A), 6-methyladenine (6 mA), 5-hydroxymethylcytosine (5hmC), guanine (G), thymine (T), and uracil (U) (see Figure 1; Supplementary Figure S5).

Particularly, it is important to distinguish between 4mC and 5 mC because they have distinct biological roles and are introduced by different methyltransferases, which can influence epigenetic regulation in completely different ways. As proof of principal we tried differentiate the two modifications, by using MS/MS fragmentation of the released nucleobases as the molecules have the same *m*/*z* ratio and the signals in the LC analysis overlap. We fragmented the nucleobases with a normalized collision energy (NCE) of 150, and unique fragments of the two bases (see Supplementary Figure S10) were plotted. Thus the method holds promise to distinguish and quantify 4mC and 5 mC together.

Furthermore, our method not only enables the detection and quantification of cytosine methylation in position five but also extends to A and 6mA, highlighting its versatility and broad applicability. We further demonstrate its sensitivity and robustness through a comparative analysis of an *E. coli* strain with low-level adenine methylation and a methylation-deficient mutant, as shown in Supplementary Figures S11–S13.

### 3.6 Method application

To apply the method in biological systems, we quantified the proportion of methylated cytosine in the analysis of DNA of the green seaweed *U. mutabilis* ‘slender’ grown in the presence or absence of its associated bacteria. In previous studies, it was observed that the growth and morphogenesis of *U. mutabilis* depend on bacterial-produced compounds such as thallusin (Wichard, 2023). In the absence of the native bacterial community, incomplete cell wall development and insufficient growth were observed (Spoerner et al., 2012). Therefore, we suspected an influence of bacterial factors on the DNA methylation of *U*. *mutabilis*.

In the presence of *U. mutabilis*-associated bacteria, the 5 mC methylation level drops significantly compared to axenic conditions (7.46% ± 0.38% vs 13.59% ± 0.77%, respectively; Figure 3C) whereas no 4 mC was detected. Overall, we found a notably high level of 5 mC methylation in these samples, within a range where enzymatic digestion of DNA can become inefficient for downstream analysis (see Figure 2D). Our findings also reveal a clear link between bacterial presence and epigenetic regulation in *U*. *mutabilis*, highlighting the role of bacterial-derived chemical mediators, such as AGMPFs, in shaping host DNA methylation patterns, which need to be further investigated.

## 4 Summary

In this study, we established a DNA hydrolysis/UHPLC-HRMS quantification protocol that enables global methylome analysis and distinguishes the key epigenetic 5-methylcytosine from cytosine as well as 6-methyladenine from adenine. It provides a rapid indication of whether a genome is globally hypo- or hypermethylated, which can be important information for various biological stages, such as during the life cycle or in disease states. It overcomes limitations of sequencing-based methods, especially the comparable high costs and the applicability to non-model organisms. Additionally, our method avoids pitfalls caused by enzymatic hydrolysis protocols. Our simple data analysis facilitates straightforward comparisons of genome-wide DNA methylation levels across various biological contexts.

A major advantage of our method is its speed and accuracy in analyzing global DNA methylation, compared to previous protocols. Moreover, our quantification approach is independent of overall methylation levels and provides reliable results even in highly methylated genomes, where enzymatic methods may fall short. Overall, our method offers a novel and reliable approach to quantitative epigenetic analysis, particularly valuable for organisms with high levels of DNA methylation, such as the seaweed *U. mutabilis.* The advantage of our global methylation screening approach is that it rapidly provides precise preliminary information on which samples warrant time-consuming sequencing. This makes the method readily applicable to medicinal and environmental screening studies, where hypo- and hypermethylated states are of particular interest, as well as in marine and terrestrial plant sciences, where the effects of environmental changes on methylation levels need to be assessed quickly.

## Data Availability

The data presented in the study are deposited in the Zenodo repository, accession number 17153000.
