# Supplementary material for: Global DNA-methylation in quantitative epigenetics: orbitrap mass spectrometry
Source: Front Mol Biosci. 2025 Sep 26;12:1681568. doi: 10.3389/fmolb.2025.1681568 (PMC12511803; doi:10.3389/fmolb.2025.1681568)
Supplement: Supplementary file 1 [file DataSheet1.pdf]

Supplementary Material

Global DNA-Methylation in Quantitative Epigenetics: Orbitrap Mass Spectrometry

Janine F. M. Otto [1], Georg Pohnert [1,2], Thomas Wichard [3], Michael Bauer [2,4] Anne Busch [2,4,5], Nico Ueberschaar [6*]

[1]Department of Instrumental Analytics/Bioorganic Analytics, Institute for Inorganic and Analytical Chemistry, Friedrich Schiller University Jena, Lessingstraße 8, 07743 Jena, Germany

[2]Cluster of Excellence Balance of the Microverse, Friedrich Schiller University Jena, Neugasse 23, 07743, Jena, Germany

[3]Chemical Ecology of Cross Kingdom Interactions, Institute for Inorganic and Analytical Chemistry, Friedrich Schiller University Jena, Lessingstraße 8, 07743 Jena, Germany

[4]Department of Anaesthesiology and Intensive Care Medicine, Jena University Hospital, Am Klinikum 1, 07743 Jena, Germany

[5]Department of Theoretical Microbial Ecology, Institute of Microbiology, Friedrich Schiller University Jena, Winzerlaerstr. 2, 07745 Jena, Germany

[6]Mass Spectrometry Platform, Friedrich Schiller University Jena, Humboldtstraße 8, 07743 Jena, Germany

*** Correspondence:**
Corresponding Author
nico.ueberschaar@uni-jena.de

Keywords: Global DNA methylation, 5-methylcytosine, Epigenetics, *Ulva mutabilis*, UHPLC-HRMS, high-throughput

JO: 0000-0002-7479-5198

GP: 0000-0003-2351-6336

TW: 0000-0003-0061-4160

MB: 0000-0002-1521-3514

AB: 0000-0001-9560-0057

NU: 0000-0002-4192-490X

Contents

Supplementary Figure 1 Deoxyformylcytidine is formed during incomplete hydrolysis with formic acid. 5

Supplementary Figure 2 Optimization of hydrolysis conditions for DNA containing cytosine. The error bars result from the standard deviation of the mean value. n = 3. 6

Supplementary Figure 3 Optimization of hydrolysis conditions for DNA containing 5-methylcytosine. The error bars result from the standard deviation of the mean value. n = 3. 6

Supplementary Figure 4 Nucleobase stability of cytosine, adenine, 5-methylcytosine, and 6-methyladenine with 2 % hydrochloric acid at 120 °C for 0 to 6 hours. n = 3. 7

Supplementary Figure 5 Chromatogram of the studied nucleobases after acid hydrolysis and separation with Phenomenex Synergi™ Fusion-RP and a gradient from a 20 mM $HCOONH_4$ aqueous solution buffered at pH 4.3 to pure acetonitrile. 7

Supplementary Figure 6 Comparison of different columns and solvents to separate different nucleobases. Hydrolysis of C, 4/5mC, T, and U is incomplete in these samples. The gradient is the same as described in the methods section. FA = formic acid. 8

Supplementary Figure 7 AUC values of cytosine after enzymatic digestion, after a combination of enzymatic and acid hydrolysis, and acid hydrolysis after acid hydrolysis are dependent only on the hydrolysis time. The error bars result from the standard deviation of the mean value. n = 3. 9

Supplementary Figure 8 Linear calibration (red line) of 5-methylcytosine used in Figure 3C. 5-methylcytosine from 0 – 10 nM with 50 nM internal standard. The light red area around the linear calibration line displays the 95% confidence interval. LOD and LOQ are given as concentration (nM) and as amount on column (fmol). n = 3. The calibration curve shown in Figure 3A was used for cytosine quantification. 10

Supplementary Figure 9 Linear calibration (red line) of cytosine, and 5-methylcytosine. Cytosine from 0 – 2.5 µM with 1.25 µM internal standard, 5-methylcytosine from 0 - 500 nM with 50 nM internal standard. The light red area around the linear calibration line displays the 95% confidence interval. LOD and LOQ are given as concentration (nM) and as amount of column (fmol). n = 3. 11

Supplementary Figure 10 MS^2 spectra of protonated 5mC and 4mC with NCE 150 at 1.30 min. The red boxes highlight the unique fragments of the methylated nucleobases. 12

Supplementary Figure 11 Comparison of the genomic DNA of *E. coli* wild-type strain DH5-α and the methyltransferase-deficient strain GM2163. The proportion of both, 6mA and mC, in E. coli GM2163 is below the LOD (0.08 and 0.14% respectively). The error bars result from the standard deviation of the mean value. n = 5. *** - $p < 0.001$. 13

Supplementary Figure 12 Linear calibration (red line, n = 3) of adenine, 0 - 100 nM with 50 nM internal standard and from 0 - 500 nM with 50 nM internal standard. The light red area around the linear calibration line displays the confidence interval. LOD = limit of detection; LOQ = limit of quantification, R^2 = coefficient of determination, $s_{x,0}$ = standard error of the mean. LOD and LOQ are given as concentration (nM, in 2 µL injection volume) and as amount on column (fmol) 13

Supplementary Figure 13 Linear calibration (red line, n = 3) of *N*-6-methyladenine, 0 - 100 nM with 50 nM internal standard and from from 0 - 500 nM with 50 nM internal standard. The light red area

around the linear calibration line displays the confidence interval. LOD = limit of detection; LOQ = limit of quantification, R^2 = coefficient of determination, $s_{x,0}$ = standard error of the mean. LOD and LOQ are given as concentration (nM, in 2 µL injection volume) and as amount on column (fmol)...14

Supplementary Figure 14 Linear calibration (red line, n = 3) of cytosine and *N*-6-methyladenine, 0 - 100 nM with 50 nM internal standard. The light red area around the linear calibration line displays the confidence interval. LOD = limit of detection; LOQ = limit of quantification, R^2 = coefficient of determination, $s_{x,0}$ = standard error of the mean. LOD and LOQ are given as concentration (nM, in 2 µL injection volume) and as amount on column (fmol). 14

Supplementary Figure 15 Linear calibration (red line, n = 3) of cytosine, adenine, 5-methylcytosine, and *N*-6-methyladenine, 0 - 5 µM with 2.5 µM internal standard. The light red area around the linear calibration line displays the confidence interval. LOD = limit of detection; LOQ = limit of quantification, R^2 = coefficient of determination, $s_{x,0}$ = standard error of the mean. LOD and LOQ are given as concentration (nM, in 2 µL injection volume) and as amount on column (fmol). 15

S**upplementary Table 1.** Theoretical and measured proportions of 5-methylcytosine (5-methylcytidine for enzymatic digestion) after acid hydrolysis or enzymatic digestion of DNA standards only containing cytosine or 5-methylcytosine mixed in different ratios. SD = standard deviation. n = 3. 16

S**upplementary Table 2.** Statistical analysis of accuracy (%Deviation) and precision (%RSD, relative standard deviation) for intra-day validation of the cytosine calibration curve from 0 to 100 nM with 50 nM internal standard, shown in Figure 3. 17

S**upplementary Table 3**. Statistical analysis of accuracy (%Deviation) and precision (%RSD, relative standard deviation) for intra-day validation of the 5-methylcytosine calibration curve from 0 to 100 nM with 50 nM internal standard, shown in Figure 3. 17

S**upplementary Table 4.** Statistical analysis of accuracy (%Deviation) and precision (%RSD, relative standard deviation) for intra-day validation of the cytosine calibration curve from 0 to 2.5 µM with 1.25 µM internal standard, shown in Supplementary Figure 9. 18

S**upplementary Table 5.** Statistical analysis of accuracy (%Deviation) and precision (%RSD, relative standard deviation) for intra-day validation of the 5-methylcytosine calibration curve from 0 to 500 nM with 50 nM internal standard, shown in Supplementary Figure 9. 18

S**upplementary Table 6.** Statistical analysis of accuracy (%Deviation) and precision (%RSD, relative standard deviation) for intra-day validation of the adenine calibration curve from 0 to 100 nM with 50 nM internal standard, shown in Supplementary Figure 13. 19

S**upplementary Table 7.** Statistical analysis of accuracy (%Deviation) and precision (%RSD, relative standard deviation) for intra-day validation of the adenine calibration curve from 0 to 500 nM with 50 nM internal standard, shown in Supplementary Figure 13. 20

S**upplementary Table 8**. Statistical analysis of accuracy (%Deviation) and precision (%RSD, relative standard deviation) for intra-day validation of the 6-methyladenine calibration curve from 0 to 100 nM with 50 nM internal standard, shown in Supplementary Figure 14. 20

S**upplementary Table 9**. Statistical analysis of accuracy (%Deviation) and precision (%RSD, relative standard deviation) for intra-day validation of the 6-methyladenine calibration curve from 0 to 500 nM with 50 nM internal standard, shown in Supplementary Figure 14. 21

Supplementary Table 10. Statistical analysis of accuracy (%Deviation) and precision (%RSD, relative standard deviation) for intra-day validation of the cytosine calibration curve from 0 to 10 nM with 50 nM internal standard, shown in Supplementary Figure 15. 21

Supplementary Table 11. Statistical analysis of accuracy (%Deviation) and precision (%RSD, relative standard deviation) for intra-day validation of the 6-methyladenine calibration curve from 0 to 10 nM with 50 nM internal standard, shown in Supplementary Figure 15. 22

Supplementary Table 12. Statistical analysis of accuracy (%Deviation) and precision (%RSD, relative standard deviation) for intra-day validation of the cytosine calibration curve from 0 to 5 µM with 2.5 µM internal standard, shown in Supplementary Figure 16. 22

Supplementary Table 13. Statistical analysis of accuracy (%Deviation) and precision (%RSD, relative standard deviation) for intra-day validation of the adenine calibration curve from 0 to 5 µM with 2.5 µM internal standard, shown in Supplementary Figure 16. 23

Supplementary Table 14. Statistical analysis of accuracy (%Deviation) and precision (%RSD, relative standard deviation) for intra-day validation of the 5-methylcytosine calibration curve from 0 to 5 µM with 2.5 µM internal standard, shown in Supplementary Figure 16. 23

Supplementary Table 15. Statistical analysis of accuracy (%Deviation) and precision (%RSD, relative standard deviation) for intra-day validation of the 6-methyladenine calibration curve from 0 to 5 µM with 2.5 µM internal standard, shown in Supplementary Figure 16. 24

Appendix 1. Biological Resources 25

Appendix 2. DNA extraction and analysis 25

Appendix 3. Reagents 25

Appendix 4. Mathematical formulas for the calculation of LOD and LOQ 25

General comment:
All experiments where results show error bars were executed in triplicates (n = 3) if not other mentioned.

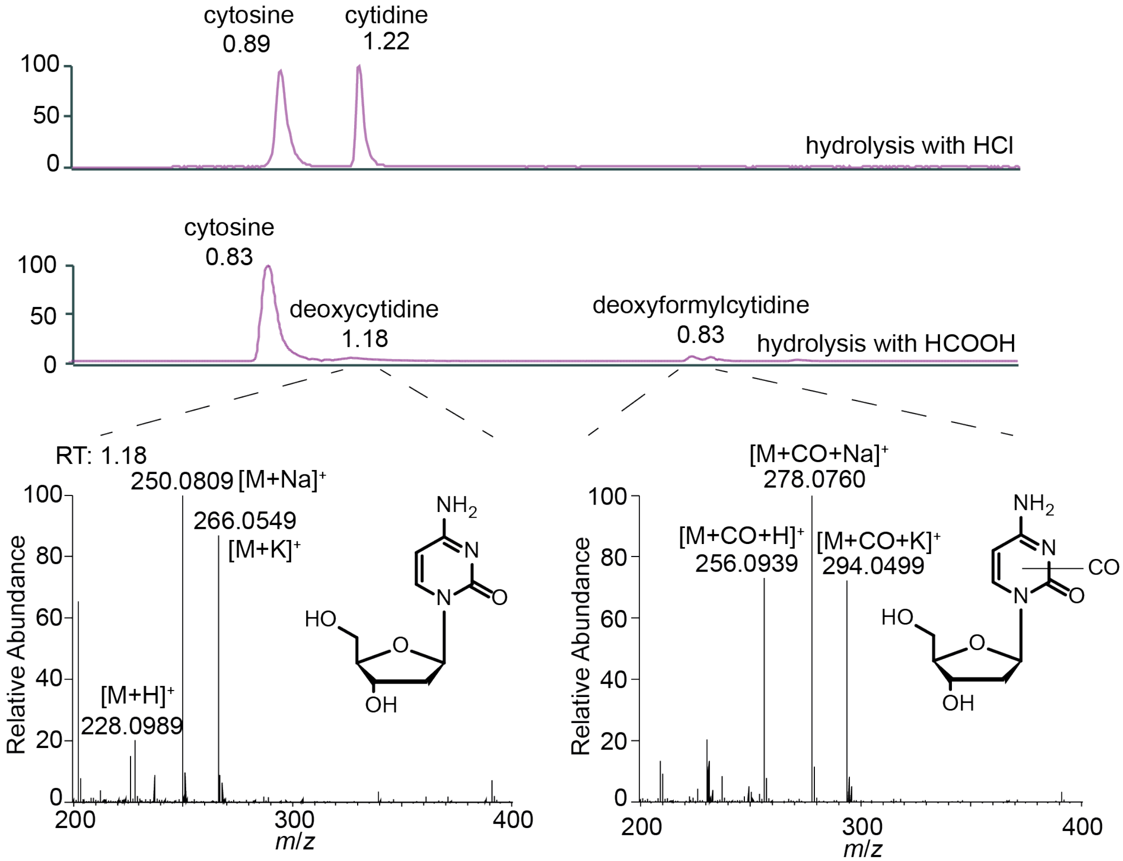

Supplementary Figure 1 Deoxyformylcytidine is formed during incomplete hydrolysis with formic acid.

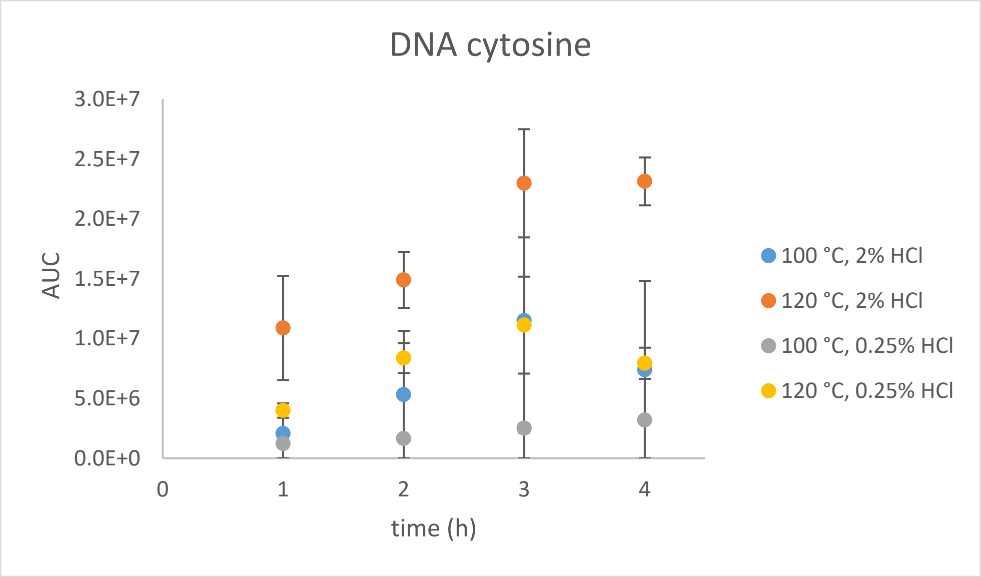

Supplementary Figure 2 Optimization of hydrolysis conditions for DNA containing cytosine. The error bars result from the standard deviation of the mean value. n = 3.

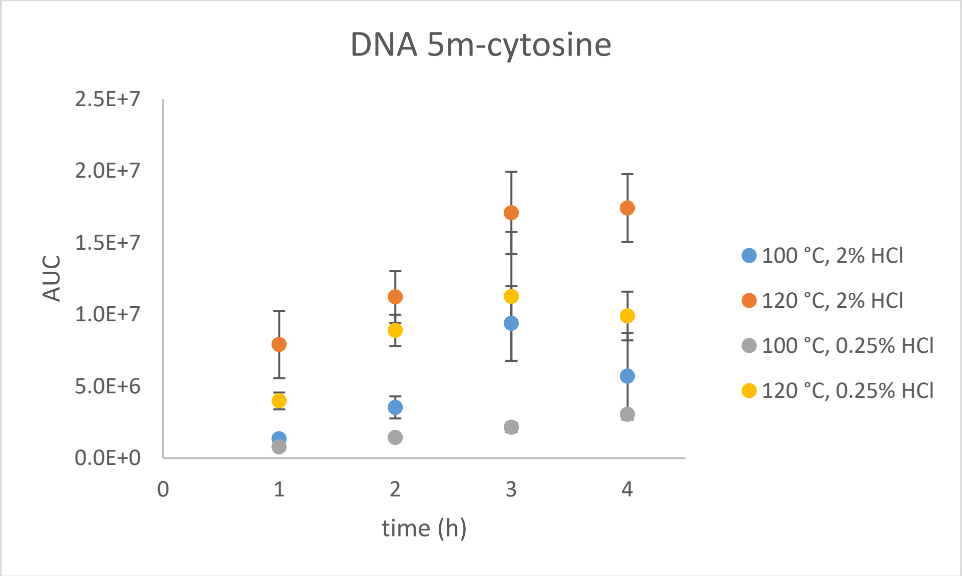

Supplementary Figure 3 Optimization of hydrolysis conditions for DNA containing 5-methylcytosine. The error bars result from the standard deviation of the mean value. n = 3.

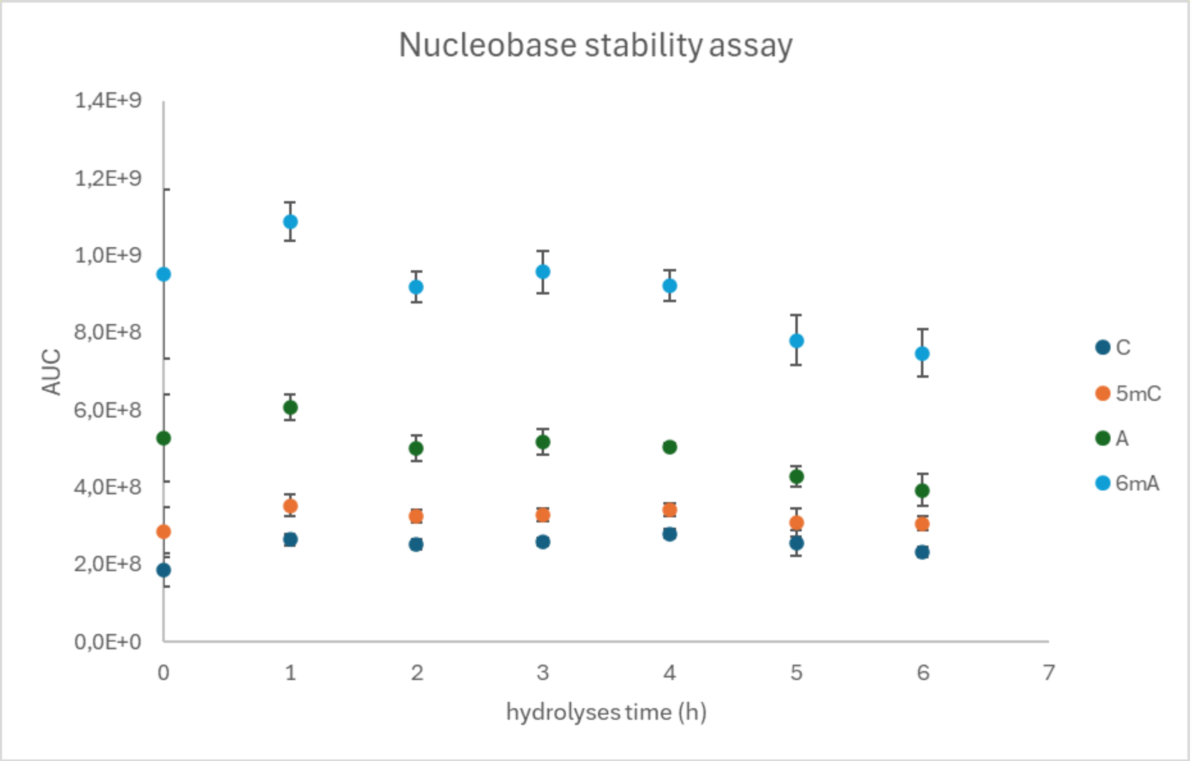

Supplementary Figure 4 Nucleobase stability of cytosine, adenine, 5-methylcytosine, and 6-methyladenine with 2 % hydrochloric acid at 120 °C for 0 to 6 hours. n = 3.

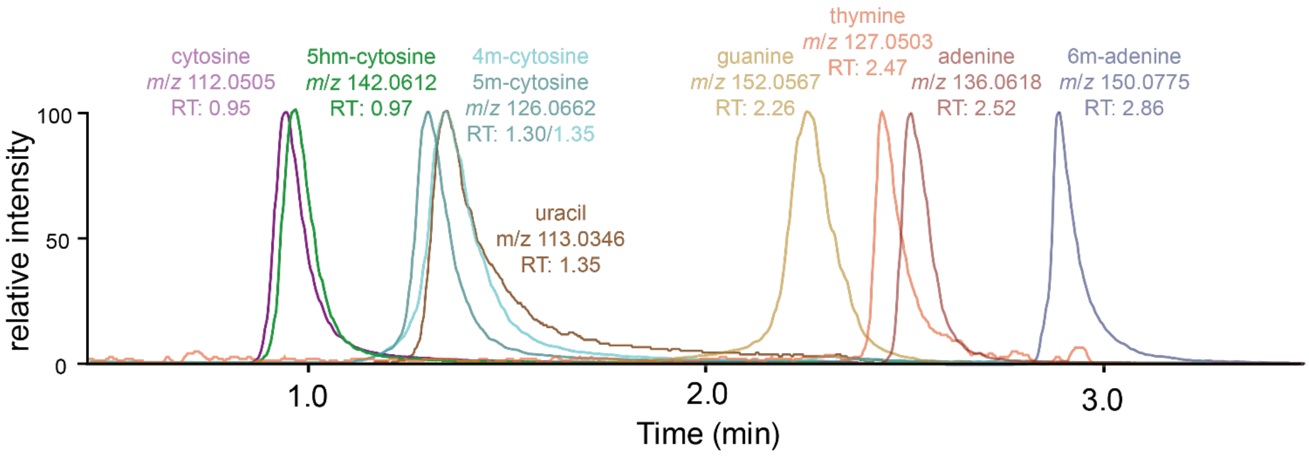

Supplementary Figure 5 Chromatogram of the studied nucleobases after acid hydrolysis and separation with Phenomenex Synergi™ Fusion-RP and a gradient from a 20 mM $HCOONH_4$ aqueous solution buffered at pH 4.3 to pure acetonitrile.

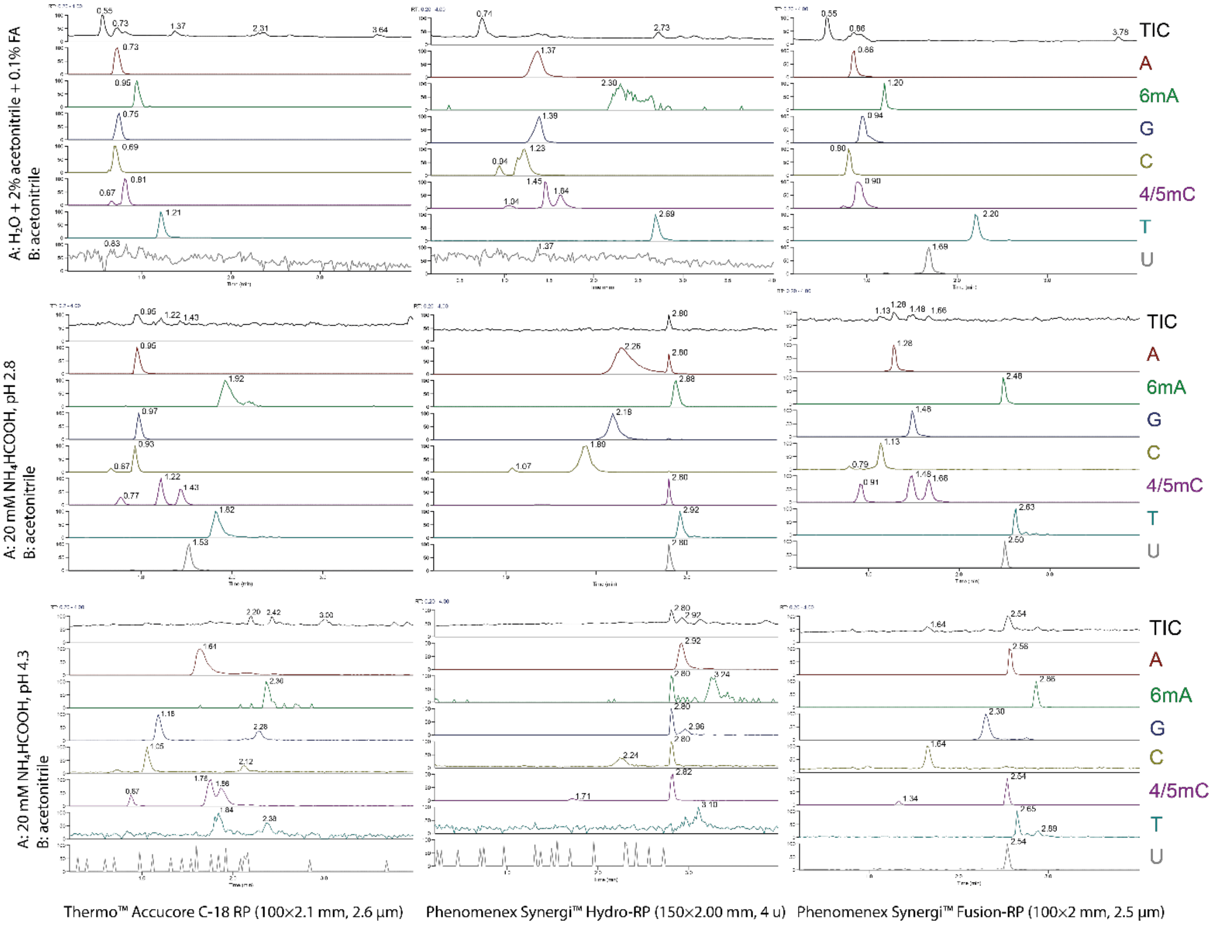

Supplementary Figure 6 Comparison of different columns and solvents to separate different nucleobases. Hydrolysis of C, 4/5mC, T, and U is incomplete in these samples. The gradient is the same as described in the methods section. FA = formic acid.

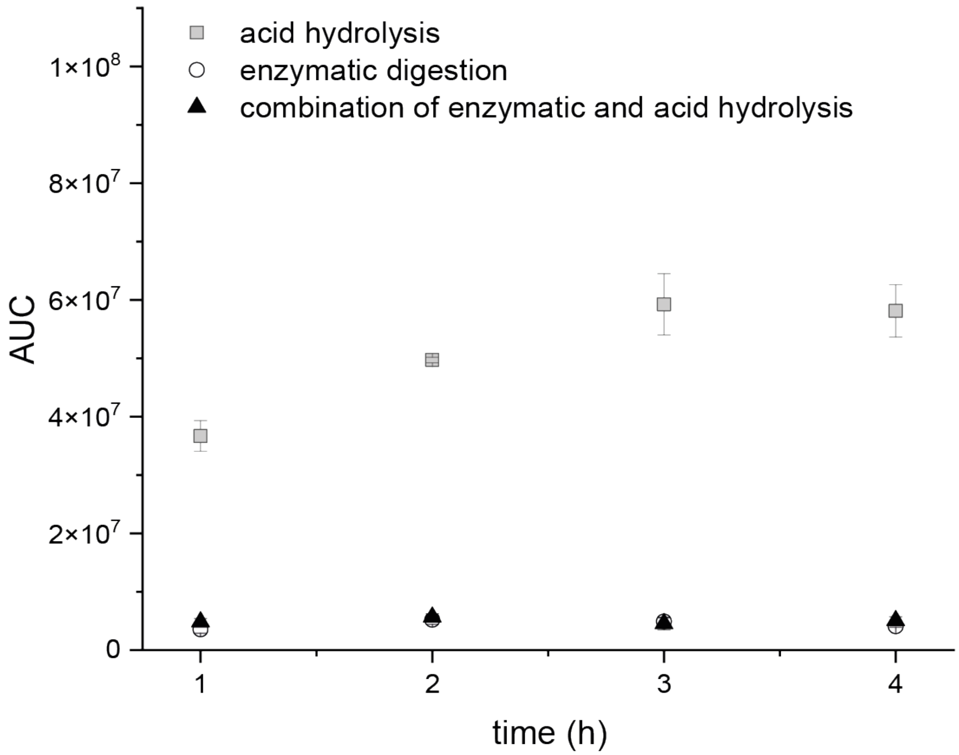

Supplementary Figure 7 AUC values of cytosine after enzymatic digestion, after a combination of enzymatic and acid hydrolysis, and acid hydrolysis after acid hydrolysis are dependent only on the hydrolysis time. The error bars result from the standard deviation of the mean value. n = 3.

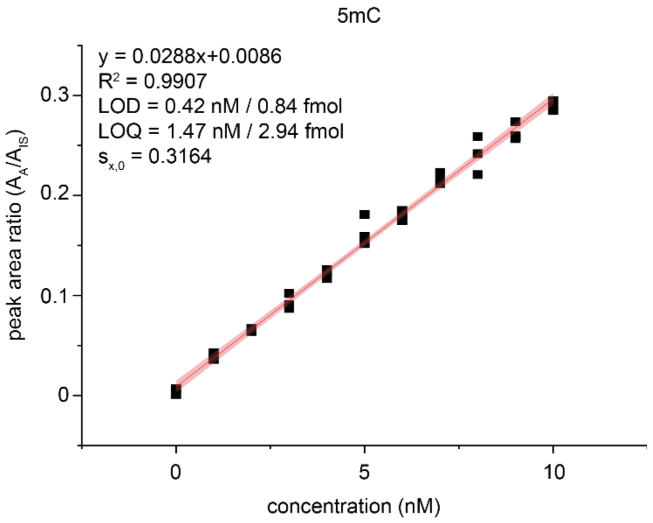

Supplementary Figure 8 Linear calibration (red line) of 5-methylcytosine used in **Figure 3C**. 5-methylcytosine from 0 – 10 nM with 50 nM internal standard. The light red area around the linear calibration line displays the 95% confidence interval. LOD and LOQ are given as concentration (nM) and as amount on column (fmol). n = 3. The calibration curve shown in **Figure 3A** was used for cytosine quantification.

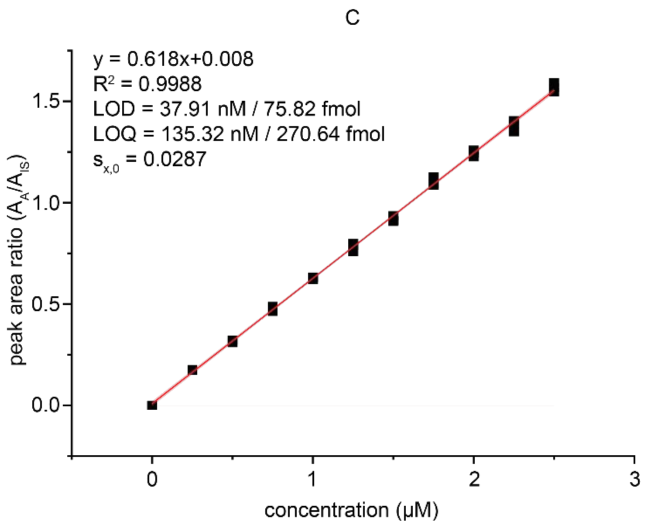

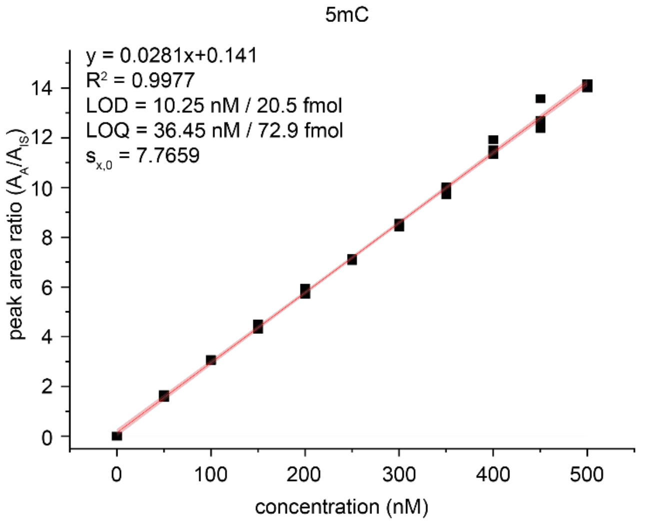

Supplementary Figure 9 Linear calibration (red line) of cytosine, and 5-methylcytosine. Cytosine from 0 – 2.5 µM with 1.25 µM internal standard, 5-methylcytosine from 0 - 500 nM with 50 nM internal standard. The light red area around the linear calibration line displays the 95% confidence interval. LOD and LOQ are given as concentration (nM) and as amount of column (fmol). n = 3.

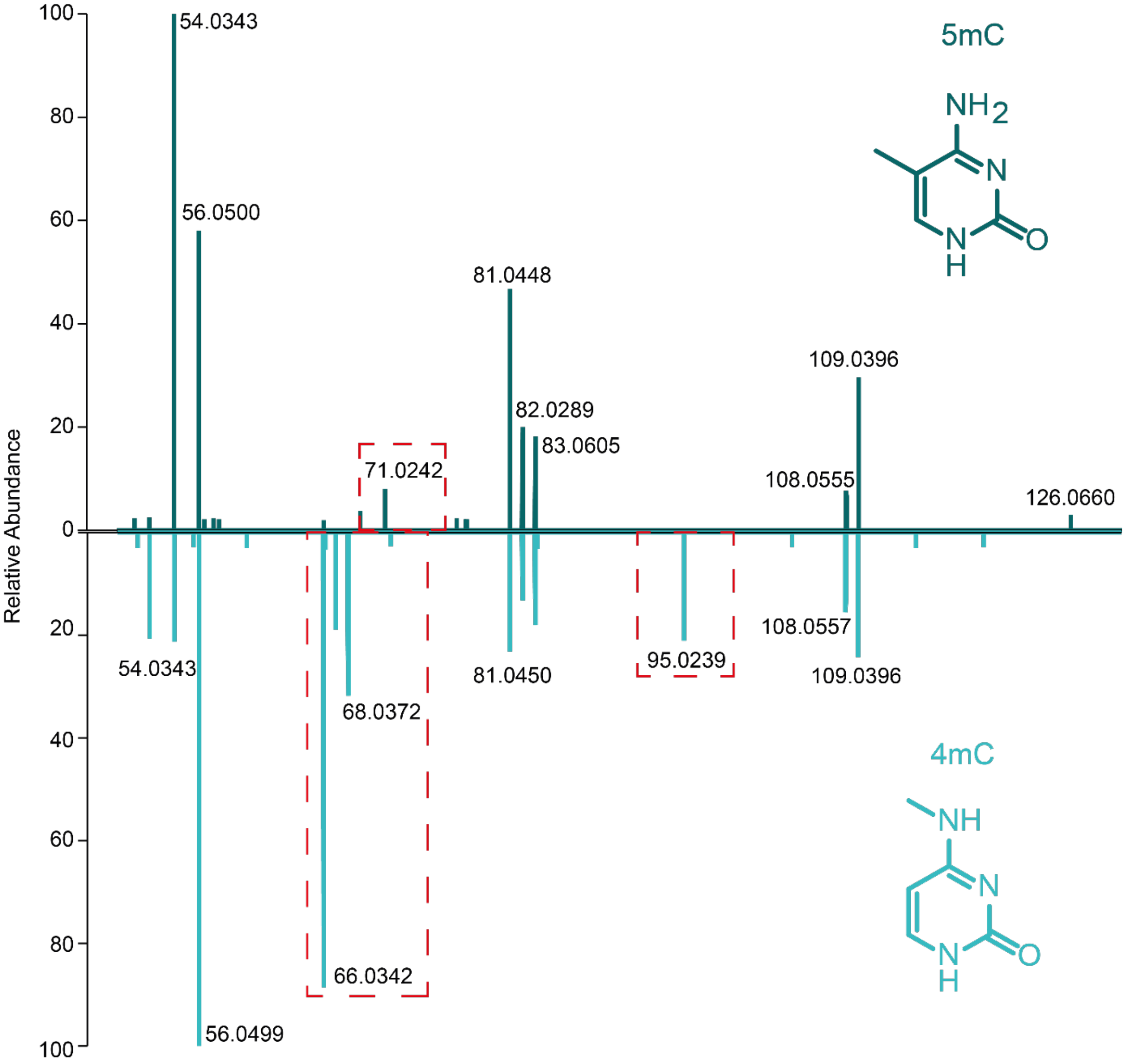

Supplementary Figure 10 MS[2] spectra of protonated 5mC and 4mC with NCE 150 at 1.30 min. The red boxes highlight the unique fragments of the methylated nucleobases.

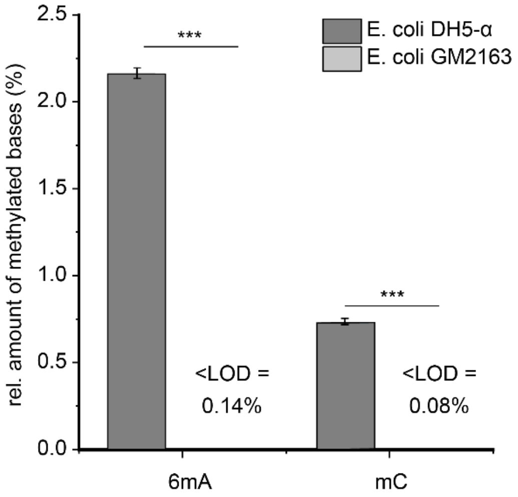

Supplementary Figure 11 Comparison of the genomic DNA of *E. coli* wild-type strain DH5-α and the methyltransferase-deficient strain GM2163. The proportion of both, 6mA and mC, in E. coli GM2163 is below the LOD (0.08 and 0.14% respectively). The error bars result from the standard deviation of the mean value. n = 5. *** - $p < 0.001$.

Adenine

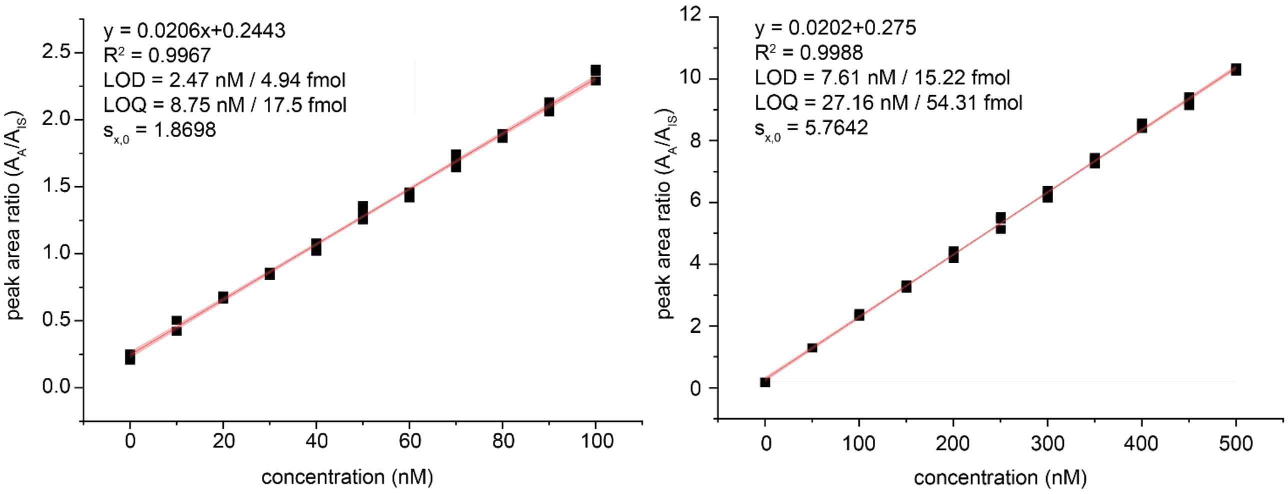

Supplementary Figure 12 Linear calibration (red line, n = 3) of adenine, 0 - 100 nM with 50 nM internal standard and from 0 - 500 nM with 50 nM internal standard. The light red area around the linear calibration line displays the confidence interval. LOD = limit of detection; LOQ = limit of quantification, R^2 = coefficient of determination, $s_{x,0}$ = standard error of the mean. LOD and LOQ are given as concentration (nM, in 2 µL injection volume) and as amount on column (fmol)

6mA

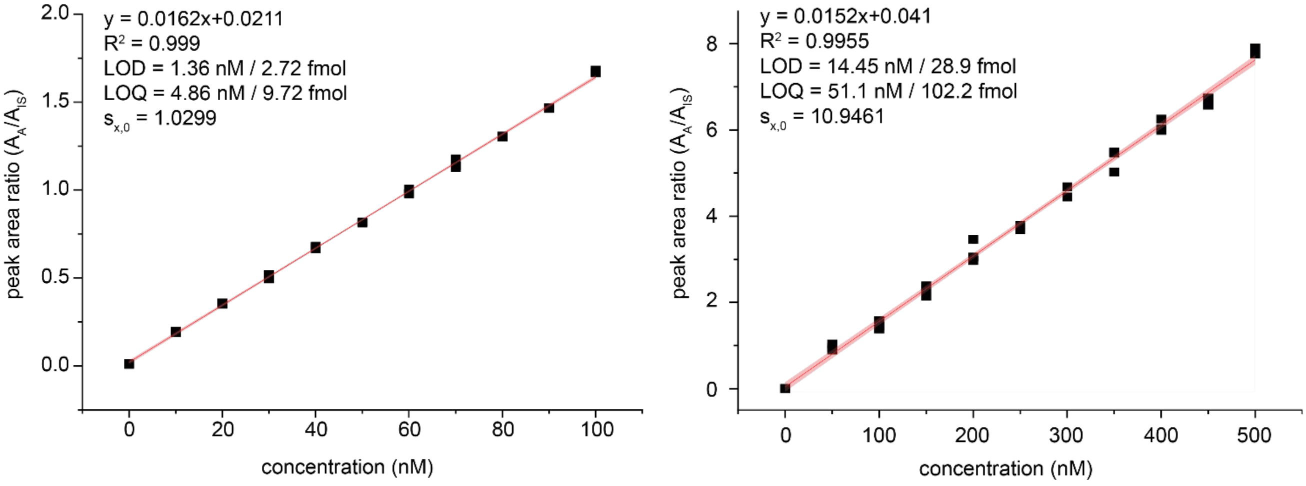

Supplementary Figure 13 Linear calibration (red line, n = 3) of *N*-6-methyladenine, 0 - 100 nM with 50 nM internal standard and from from 0 - 500 nM with 50 nM internal standard. The light red area around the linear calibration line displays the confidence interval. LOD = limit of detection; LOQ = limit of quantification, R^2 = coefficient of determination, $s_{x,0}$ = standard error of the mean. LOD and LOQ are given as concentration (nM, in 2 µL injection volume) and as amount on column (fmol).

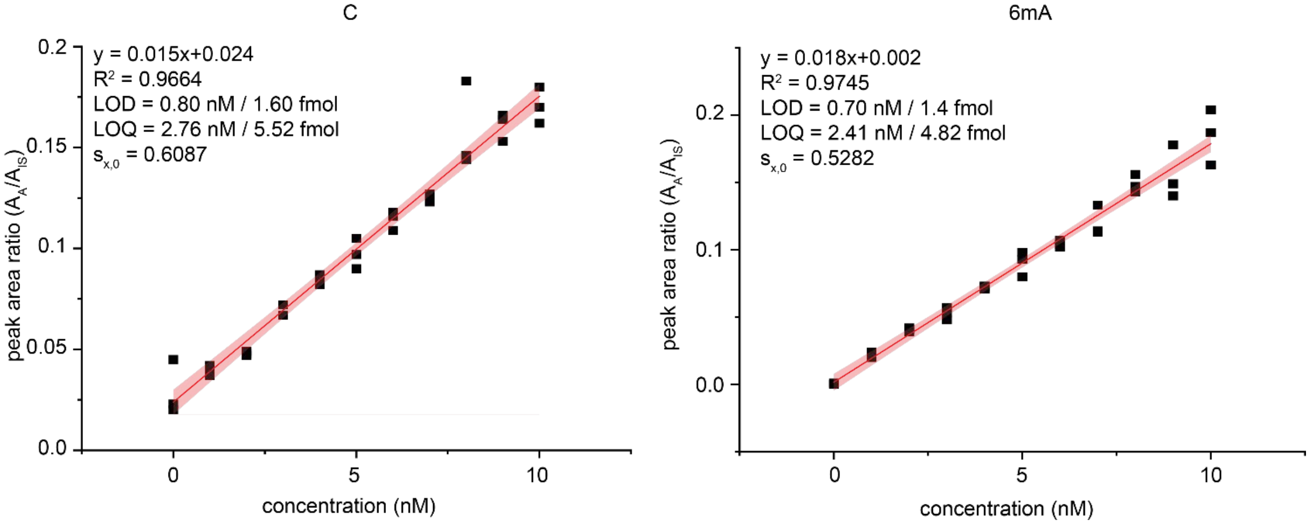

Supplementary Figure 14 Linear calibration (red line, n = 3) of cytosine and *N*-6-methyladenine, 0 - 100 nM with 50 nM internal standard. The light red area around the linear calibration line displays the confidence interval. LOD = limit of detection; LOQ = limit of quantification, R^2 = coefficient of

determination, $s_{x,0}$ = standard error of the mean. LOD and LOQ are given as concentration (nM, in 2 µL injection volume) and as amount on column (fmol).

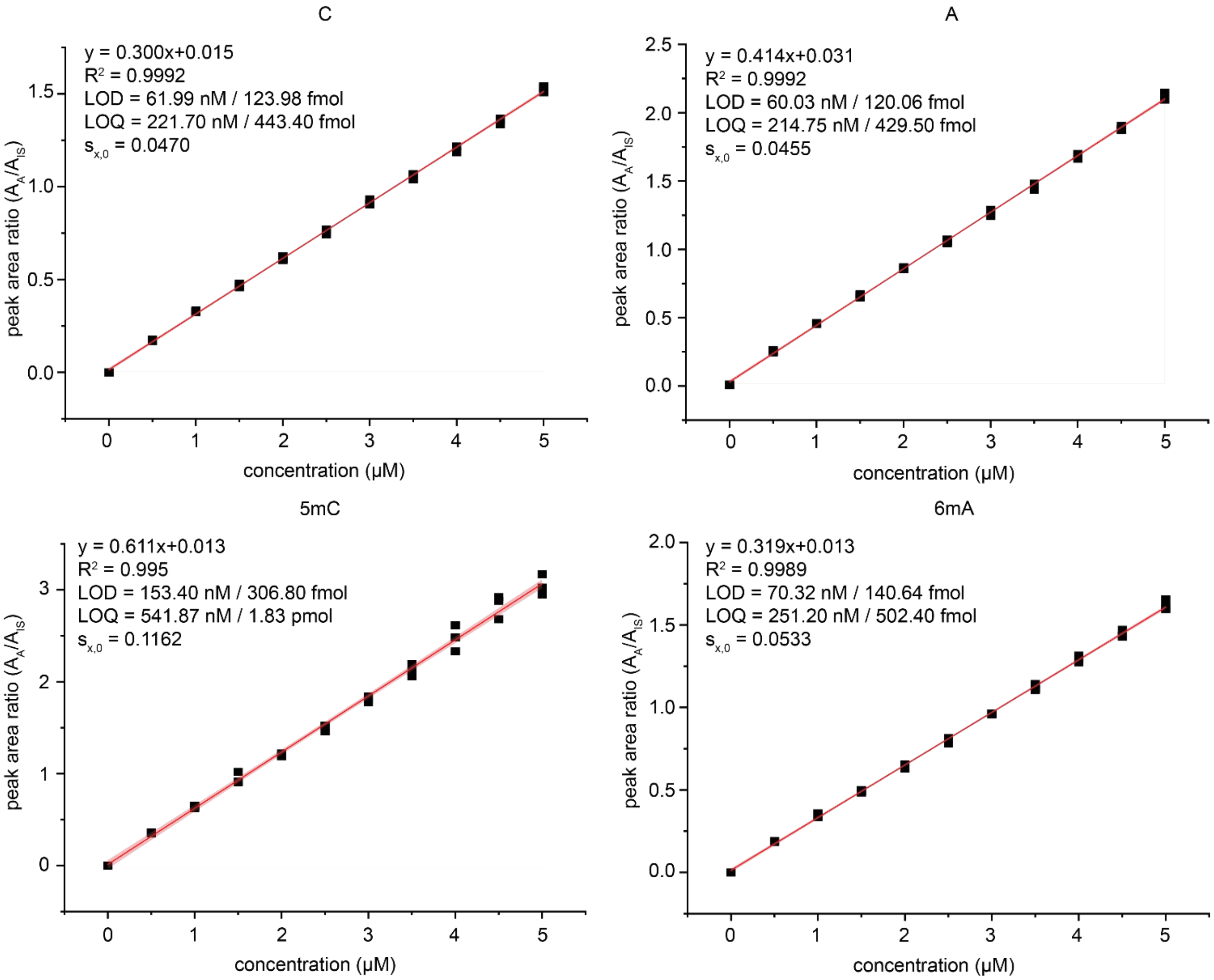

Supplementary Figure 15 Linear calibration (red line, n=3) of cytosine, adenine, 5-methylcytosine, and *N*-6-methyladenine, 0 - 5 µM with 2.5 µM internal standard. The light red area around the linear calibration line displays the confidence interval. LOD = limit of detection; LOQ = limit of quantification, R^2 = coefficient of determination, $s_{x,0}$ = standard error of the mean. LOD and LOQ are given as concentration (nM, in 2 µL injection volume) and as amount on column (fmol).

Supplementary Table 1. Theoretical and measured proportions of 5-methylcytosine (5-methylcytidine for enzymatic digestion) after acid hydrolysis or enzymatic digestion of DNA standards only containing cytosine or 5-methylcytosine mixed in different ratios. SD = standard deviation. n = 3.

Theoretical values (%)	Acid hydrolysis		Enzymatic digestion	
	Measured values (%)	SD	Measured values (%)	SD
100	97.59	0.36	100	0
50	42.21	0.81	21.90	9.15
10	7.83	0.41	8.63	3.59
1	1.94	0.09	0	0
0.1	0.87	0.01	0	0
0	0.57	0.08	0	0

Supplementary Table 2. Statistical analysis of accuracy (%Deviation) and precision (%RSD, relative standard deviation) for intra-day validation of the cytosine calibration curve from 0 to 100 nM with 50 nM internal standard, shown in **Figure 3**.

Level	Nominal concentration (nM)	Rep1	Rep2	Rep3	Mean measured	SD	%RSD	%Deviation	Accuracy acceptance
1	10	10.72	10.72	11.43	10.96	0.405	3.70	9.58	PASS
2	20	19.85	20.55	21.25	20.55	0.702	3.41	2.73	PASS
3	30	30.37	29.67	30.37	30.13	0.405	1.34	0.45	PASS
4	40	38.79	40.89	40.19	39.96	1.072	2.68	-0.11	PASS
5	50	49.31	52.82	50.01	50.71	1.856	3.66	1.43	PASS
6	60	57.73	58.43	59.83	58.67	1.072	1.83	-2.22	PASS
7	70	68.25	69.66	71.06	69.66	1.403	2.01	-0.49	PASS
8	80	76.67	79.48	80.18	78.78	1.856	2.36	-1.53	PASS
9	90	87.90	88.60	94.21	90.24	3.461	3.84	0.26	PASS
10	100	100.53	99.12	104.03	101.23	2.530	2.50	1.23	PASS

Supplementary Table 3. Statistical analysis of accuracy (%Deviation) and precision (%RSD, relative standard deviation) for intra-day validation of the 5-methylcytosine calibration curve from 0 to 100 nM with 50 nM internal standard, shown in **Figure 3**.

Level	Nominal concentration	Rep1	Rep2	Rep3	Mean measured	SD	%RSD	%Deviation	Accuracy acceptance
1	10	10.78	11.90	11.55	11.41	0.572	5.01	14.09	PASS
2	20	19.86	19.37	20.66	19.97	0.652	3.27	-0.17	PASS
3	30	28.17	29.57	34.01	30.58	3.045	9.96	1.94	PASS
4	40	37.78	39.35	39.77	38.96	1.049	2.69	-2.59	PASS
5	50	50.14	51.50	49.72	50.46	0.931	1.85	0.91	PASS
6	60	59.61	61.67	61.56	60.94	1.161	1.90	1.57	PASS

7	70	68.41	69.04	71.34	69.59	1.545	2.22	-0.58	PASS
8	80	71.41	78.53	80.35	76.77	4.726	6.16	-4.04	PASS
9	90	88.38	90.27	93.69	90.78	2.691	2.96	0.87	PASS
10	100	108.22	95.16	101.06	101.48	6.541	6.45	1.48	PASS

Supplementary Table 4. Statistical analysis of accuracy (%Deviation) and precision (%RSD, relative standard deviation) for intra-day validation of the cytosine calibration curve from 0 to 2.5 µM with 1.25 µM internal standard, shown in **Supplementary Figure 9**.

Level	Nominal concentration	Rep1	Rep2	Rep3	Mean measured	SD	%RSD	%Deviation	Accuracy acceptance
1	0.25	0.27	0.27	0.27	0.27	0.004	1.52	7.51	PASS
2	0.5	0.49	0.51	0.51	0.50	0.012	2.45	0.14	PASS
3	0.75	0.74	0.74	0.78	0.75	0.023	3.06	0.20	PASS
4	1	1.01	0.99	1.00	1.00	0.010	0.97	0.18	PASS
5	1.25	1.21	1.28	1.26	1.25	0.035	2.82	0.08	PASS
6	1.5	1.45	1.50	1.46	1.47	0.025	1.70	-1.82	PASS
7	1.75	1.81	1.74	1.76	1.77	0.035	1.97	1.20	PASS
8	2	1.97	2.00	2.02	2.00	0.027	1.34	-0.07	PASS
9	2.25	2.17	2.23	2.26	2.22	0.045	2.04	-1.30	PASS
10	2.5	2.49	2.52	2.56	2.53	0.036	1.44	1.02	PASS

Supplementary Table 5. Statistical analysis of accuracy (%Deviation) and precision (%RSD, relative standard deviation) for intra-day validation of the 5-methylcytosine calibration curve from 0 to 500 nM with 50 nM internal standard, shown in **Supplementary Figure 9.**

Level	Nominal concentration	Rep1	Rep2	Rep3	Mean measured	SD	%RSD	%Deviation	Accuracy acceptance
1	50	50.32	54.69	52.10	52.37	2.198	4.20	4.74	PASS
2	100	103.19	103.90	104.61	103.90	0.711	0.68	3.90	PASS
3	150	155.14	148.32	152.80	152.09	3.466	2.28	1.39	PASS

4	200	197.64	206.91	198.10	200.88	5.226	2.60	0.44	PASS
5	250	245.89	247.81	249.13	247.61	1.626	0.66	-0.96	PASS
6	300	293.97	293.58	299.51	295.69	3.319	1.12	-1.44	PASS
7	350	340.02	348.26	351.21	346.50	5.801	1.67	-1.00	PASS
8	400	397.51	418.83	404.40	406.91	10.879	2.67	1.73	PASS
9	450	446.37	434.50	477.07	452.65	21.967	4.85	0.59	PASS
10	500	498.42	492.84	497.71	496.33	3.037	0.61	-0.73	PASS

Supplementary Table 6. Statistical analysis of accuracy (%Deviation) and precision (%RSD, relative standard deviation) for intra-day validation of the adenine calibration curve from 0 to 100 nM with 50 nM internal standard, shown in **Supplementary Figure 13.**

Level	Nominal concentration	Rep1	Rep2	Rep3	Mean measured	SD	%RSD	%Deviation	Accuracy acceptance
1	10	8.61	12.58	12.48	11.22	2.266	20.19	12.21	PASS
2	20	20.18	20.96	21.44	20.86	0.635	3.05	4.30	PASS
3	30	28.80	29.97	29.29	29.35	0.584	1.99	-2.16	PASS
4	40	39.80	40.62	37.52	39.31	1.606	4.08	-1.71	PASS
5	50	51.67	54.14	48.86	51.55	2.642	5.12	3.11	PASS
6	60	58.83	56.80	58.98	58.20	1.219	2.09	-2.99	PASS
7	70	67.75	72.64	70.02	70.14	2.448	3.49	0.20	PASS
8	80	80.15	78.79	78.26	79.07	0.974	1.23	-1.17	PASS
9	90	88.82	87.85	91.53	89.40	1.908	2.13	-0.67	PASS
10	100	99.09	103.44	102.48	101.67	2.289	2.25	1.67	PASS

Supplementary Table 7. Statistical analysis of accuracy (%Deviation) and precision (%RSD, relative standard deviation) for intra-day validation of the adenine calibration curve from 0 to 500 nM with 50 nM internal standard, shown in **Supplementary Figure 13.**

Level	Nominal concentration	Rep1	Rep2	Rep3	Mean measured	SD	%RSD	%Deviation	Accuracy acceptance
1	50	50.73	50.19	51.03	50.65	0.427	0.84	1.30	PASS
2	100	102.76	100.92	105.78	103.15	2.452	2.38	3.15	PASS
3	150	151.61	146.11	150.27	149.33	2.868	1.92	-0.45	PASS
4	200	193.92	202.60	206.26	200.93	6.336	3.15	0.46	PASS
5	250	257.34	240.60	260.96	252.97	10.865	4.29	1.19	PASS
6	300	290.24	302.83	295.20	296.09	6.340	2.14	-1.30	PASS
7	350	345.49	351.68	355.84	351.01	5.211	1.48	0.29	PASS
8	400	411.39	402.22	407.42	407.01	4.597	1.13	1.75	PASS
9	450	438.98	446.66	453.55	446.40	7.287	1.63	-0.80	PASS
10	500	494.18	496.81	499.78	496.92	2.801	0.56	-0.62	PASS

Supplementary Table 8. Statistical analysis of accuracy (%Deviation) and precision (%RSD, relative standard deviation) for intra-day validation of the 6-methyladenine calibration curve from 0 to 100 nM with 50 nM internal standard, shown in **Supplementary Figure 14.**

Level	Nominal concentration	Rep1	Rep2	Rep3	Mean measured	SD	%RSD	%Deviation	Accuracy acceptance
1	10	10.86	10.43	10.98	10.75	0.292	2.71	7.54	PASS
2	20	20.79	20.86	20.36	20.67	0.269	1.30	3.35	PASS
3	30	29.31	30.79	30.54	30.22	0.794	2.63	0.72	PASS
4	40	40.73	39.86	40.60	40.40	0.467	1.16	1.00	PASS
5	50	48.87	48.87	49.24	49.00	0.214	0.44	-2.00	PASS
6	60	59.00	59.30	60.85	59.72	0.992	1.66	-0.47	PASS
7	70	68.38	68.93	71.34	69.55	1.575	2.26	-0.64	PASS
8	80	79.42	78.99	79.30	79.24	0.223	0.28	-0.95	PASS
9	90	89.05	89.17	89.36	89.20	0.155	0.17	-0.89	PASS

10	100	101.52	102.51	101.64	101.89	0.538	0.53	1.89	PASS

Supplementary Table 9. Statistical analysis of accuracy (%Deviation) and precision (%RSD, relative standard deviation) for intra-day validation of the 6-methyladenine calibration curve from 0 to 500 nM with 50 nM internal standard, shown in **Supplementary Figure 14.**

Level	Nominal concentration	Rep1	Rep2	Rep3	Mean measured	SD	%RSD	%Deviation	Accuracy acceptance
1	50	65.78	60.50	56.55	60.94	4.631	7.60	21.88	FALSE
2	100	93.40	88.66	101.18	94.41	6.324	6.70	-5.59	PASS
3	150	149.38	138.43	154.52	147.45	8.216	5.57	-1.70	PASS
4	200	198.56	193.16	225.53	205.75	17.342	8.43	2.88	PASS
5	250	246.76	244.12	240.10	243.66	3.353	1.38	-2.54	PASS
6	300	306.30	290.61	306.10	301.00	9.003	2.99	0.33	PASS
7	350	357.46	359.84	328.78	348.69	17.285	4.96	-0.37	PASS
8	400	392.21	409.42	394.85	398.82	9.268	2.32	-0.29	PASS
9	450	439.88	430.91	441.79	437.53	5.808	1.33	-2.77	PASS
10	500	518.21	508.91	515.30	514.14	4.756	0.93	2.83	PASS

Supplementary Table 10. Statistical analysis of accuracy (%Deviation) and precision (%RSD, relative standard deviation) for intra-day validation of the cytosine calibration curve from 0 to 10 nM with 50 nM internal standard, shown in **Supplementary Figure 15**.

Level	Nominal concentration	Rep1	Rep2	Rep3	Mean measured	SD	%RSD	%Deviation	Accuracy acceptance
1	1	0.87	0.93	1.20	1.00	0.175	17.44	0.08	PASS
2	2	1.66	1.53	1.59	1.59	0.066	4.14	-20.27	FALSE
3	3	3.18	2.85	2.85	2.96	0.190	6.44	-1.39	PASS
4	4	3.97	4.17	3.84	3.99	0.166	4.16	-0.19	PASS
5	5	4.83	5.36	4.37	4.85	0.495	10.21	-3.00	PASS
6	6	5.62	6.08	6.21	5.97	0.312	5.22	-0.47	PASS
7	7	6.54	6.74	6.81	6.70	0.137	2.05	-4.32	PASS
8	8	8.06	10.50	7.93	8.83	1.449	16.41	10.39	PASS

9	9	9.25	8.52	9.38	9.05	0.462	5.10	0.57	PASS
10	10	9.65	9.12	10.30	9.69	0.595	6.14	-3.11	PASS

Supplementary Table 11. Statistical analysis of accuracy (%Deviation) and precision (%RSD, relative standard deviation) for intra-day validation of the 6-methyladenine calibration curve from 0 to 10 nM with 50 nM internal standard, shown in **Supplementary Figure 15.**

Level	Nominal concentration	Rep1	Rep2	Rep3	Mean measured	SD	%RSD	%Deviation	Accuracy acceptance
1	1	1.15	1.04	1.26	1.15	0.113	9.81	15.20	PASS
2	2	2.23	2.28	2.11	2.21	0.086	3.91	10.32	PASS
3	3	2.90	2.62	3.13	2.88	0.255	8.83	-3.86	PASS
4	4	3.98	3.92	4.03	3.98	0.056	1.42	-0.59	PASS
5	5	5.45	5.16	4.43	5.01	0.525	10.47	0.24	PASS
6	6	5.78	5.67	5.95	5.80	0.142	2.45	-3.29	PASS
7	7	6.29	6.35	7.42	6.69	0.637	9.52	-4.46	PASS
8	8	7.99	8.21	8.72	8.31	0.376	4.53	3.84	PASS
9	9	9.96	8.33	7.82	8.70	1.122	12.89	-3.31	PASS
10	10	9.12	10.47	11.43	10.34	1.164	11.25	3.41	PASS

Supplementary Table 12. Statistical analysis of accuracy (%Deviation) and precision (%RSD, relative standard deviation) for intra-day validation of the cytosine calibration curve from 0 to 5 µM with 2.5 µM internal standard, shown in **Supplementary Figure 16.**

Level	Nominal concentration	Rep1	Rep2	Rep3	Mean measured	SD	%RSD	%Deviation	Accuracy acceptance
1	0.5	0.52	0.53	0.54	0.53	0.012	2.21	5.90	PASS
2	1	1.06	1.04	1.05	1.05	0.013	1.29	4.80	PASS
3	1.5	1.48	1.50	1.55	1.51	0.035	2.30	0.50	PASS
4	2	1.97	1.99	2.04	2.00	0.032	1.62	0.08	PASS
5	2.5	2.43	2.48	2.52	2.48	0.043	1.75	-0.98	PASS

6	3	2.97	2.99	3.06	3.01	0.049	1.62	0.21	PASS
7	3.5	3.43	3.51	3.52	3.48	0.050	1.44	-0.47	PASS
8	4	3.91	4.01	4.01	3.98	0.060	1.50	-0.53	PASS
9	4.5	4.41	4.51	4.48	4.47	0.051	1.14	-0.68	PASS
10	5	4.99	5.06	5.10	5.05	0.056	1.11	0.98	PASS

Supplementary Table 13. Statistical analysis of accuracy (%Deviation) and precision (%RSD, relative standard deviation) for intra-day validation of the adenine calibration curve from 0 to 5 µM with 2.5 µM internal standard, shown in **Supplementary Figure 16.**

Level	Nominal concentration	Rep1	Rep2	Rep3	Mean measured	SD	%RSD	%Deviation	Accuracy acceptance
1	0.5	0.52	0.54	0.56	0.54	0.018	3.38	8.00	PASS
2	1	1.03	1.04	1.03	1.03	0.007	0.68	3.21	PASS
3	1.5	1.49	1.53	1.55	1.52	0.030	1.96	1.66	PASS
4	2	1.99	2.00	2.03	2.01	0.017	0.87	0.33	PASS
5	2.5	2.45	2.48	2.52	2.48	0.033	1.32	-0.73	PASS
6	3	2.93	2.97	3.04	2.98	0.056	1.87	-0.60	PASS
7	3.5	3.40	3.48	3.50	3.46	0.054	1.56	-1.09	PASS
8	4	3.95	3.99	4.03	3.99	0.040	1.00	-0.28	PASS
9	4.5	4.45	4.53	4.46	4.48	0.039	0.88	-0.41	PASS
10	5	4.99	5.05	5.11	5.05	0.059	1.17	1.04	PASS

Supplementary Table 14. Statistical analysis of accuracy (%Deviation) and precision (%RSD, relative standard deviation) for intra-day validation of the 5-methylcytosine calibration curve from 0 to 5 µM with 2.5 µM internal standard, shown in **Supplementary Figure 16.**

Level	Nominal concentration	Rep1	Rep2	Rep3	Mean measured	SD	%RSD	%Deviation	Accuracy acceptance
1	0.5	0.56	0.57	0.55	0.56	0.010	1.75	12.16	PASS
2	1	1.00	1.05	1.04	1.03	0.026	2.53	3.12	PASS
3	1.5	1.46	1.48	1.65	1.53	0.102	6.69	1.89	PASS
4	2	1.93	1.95	1.98	1.95	0.023	1.20	-2.44	PASS

5	2.5	2.37	2.47	2.47	2.44	0.055	2.27	-2.52	PASS
6	3	2.89	2.94	3.00	2.94	0.052	1.78	-1.90	PASS
7	3.5	3.35	3.57	3.44	3.46	0.110	3.19	-1.28	PASS
8	4	3.80	4.26	4.05	4.03	0.234	5.79	0.87	PASS
9	4.5	4.76	4.37	4.70	4.61	0.210	4.56	2.42	PASS
10	5	4.81	5.17	4.93	4.97	0.183	3.69	-0.61	PASS

Supplementary Table 15. Statistical analysis of accuracy (%Deviation) and precision (%RSD, relative standard deviation) for intra-day validation of the 6-methyladenine calibration curve from 0 to 5 µM with 2.5 µM internal standard, shown in **Supplementary Figure 16.**

Level	Nominal concentration	Rep1	Rep2	Rep3	Mean measured	SD	%RSD	%Deviation	Accuracy acceptance
1	0.5	0.55	0.54	0.56	0.55	0.008	1.51	9.67	PASS
2	1	1.01	1.04	1.08	1.04	0.035	3.32	4.49	PASS
3	1.5	1.48	1.51	1.52	1.50	0.023	1.50	0.32	PASS
4	2	1.93	2.00	2.01	1.98	0.042	2.12	-0.87	PASS
5	2.5	2.41	2.51	2.49	2.47	0.052	2.11	-1.21	PASS
6	3	2.96	2.97	2.98	2.97	0.009	0.32	-0.92	PASS
7	3.5	3.43	3.46	3.54	3.48	0.058	1.68	-0.71	PASS
8	4	3.95	4.08	4.02	4.02	0.063	1.56	0.42	PASS
9	4.5	4.44	4.57	4.46	4.49	0.071	1.59	-0.22	PASS
10	5	4.97	4.99	5.15	5.04	0.099	1.96	0.72	PASS

Biological Resources: *E. coli* DH5α (fhuA2 lac(del)U169 phoA glnV44 Φ80' lacZ(del)M15 gyrA96 recA1 relA1 endA1 thi-1 hsdR17) and the methyltransferase negative strain *E. coli* GM2163 (araC14, leuB6(Am), fhuA13, lacY1, tsx-78, glnX44(AS), galK2(Oc), galT22, λ^-, mcrA0, dcm-6, hisG4(Oc), rfbC1, rpsL136(strR), dam-13::Tn9, xylA5, mtl-1, thiE1, mcrB9999, hsdR2) were cultured in Luria-Bertani (LB) medium (Oxoid, Germany). For overnight precultures, single colonies were picked from freshly streaked LB agar plates and inoculated into 5 mL LB-Amp medium in sterile 15 mL culture tubes. Cultures were incubated for 16–18 hours at 37 °C with shaking at 200 rpm in a rotary incubator. All bacterial cultures were prepared under sterile conditions and visually inspected for turbidity to ensure appropriate growth prior to downstream processing.

DNA extraction and analysis: *E. coli* genomic DNA was isolated from 16–18 h cultures of using a commercial genomic DNA purification kit according to the PureLink DNA Mini Kit (Thermo Fisher Scientific, Invitrogen) protocol, with adaptations for Gram-negative bacteria as specified in the instructions. Briefly, 1 mL of each bacterial culture was harvested by centrifugation at 8,000 × g for 5 minutes at room temperature. The supernatant was discarded, and the bacterial pellet was resuspended in 180 µL of lysis buffer. For *E. coli*, this consisted of buffer A supplemented with 20 µL proteinase K (20 mg/mL), followed by incubation at 56 °C for 30 minutes with occasional vortexing. RNase A (typically 20 µg in 20 µL, as supplied by the kit, RNA-free preparation was confirmed by monitoring of uracil, which was not detected) was added during the lysis step to remove contaminating RNA. After complete lysis, samples were processed according to the standard binding, washing, and elution steps using silica membrane columns, with final elution of genomic DNA in 50–100 µL nuclease-free water (a step that proved essential). DNA quantity and purity were assessed using spectrophotometric measurement, and DNA was stored at −20 °C until further analysis.

Reagents: Chemicals were purchased from the following suppliers: 2'-deoxy-*N*-6-methyladenosine-d^3 ($6mAd^3$), 2'-deoxyadenosine-$^{15}N_5$ (A-$^{15}N_5$, Cambridge Isotope Laboratories, Inc., Tewksbury, MA, US) were dissolved in water and used as internal standards (IS). 2'-deoxy-*N*-6-methyladenosine (Sigma-Aldrich Chemie GmbH, Taufkirchen, Germany), and 2'-deoxyadenosine (TCI Deutschland GmbH, Eschborn, Germany) were used for the optimization of the hydrolysis method. Adenine, cytosine, 5-methylcytosine, guanine, thymine and uracil were purchased from Sigma-Aldrich Chemie GmbH, Taufkirchen, Germany, and 6-methyladenine was purchased from TargetMol, BIOZOL Diagnostica Vertrieb GmbH, Hamburg, Germany.

Mathematical formulas for the calculation of LOD and LOQ on the basis of a linear model:

$y = a + bx$; n: number of measurements (here n = 33), $s_{y,x}$: residual standard deviation, $s_{x,0}$: process standard deviation, f : degrees of freedom (f = n − 2), α: probability of error (here 0.05 = 95%), y_x: critical signal, m: measurements per sample (here m = 1)

$$Q_{xx} = \sum_{i=1}^{n} x_i^2 - \frac{\left(\sum_{i=1}^{n} x_i\right)^2}{n}$$

$$Q_{yy} = \sum_{i=1}^{n} y_i^2 - \frac{\left(\sum_{i=1}^{n} y_i\right)^2}{n}$$

$$Q_{xy} = \sum_{i=1}^{n} (x_i y_i) - \frac{\sum_{i=1}^{n} x_i \sum_{i=1}^{n} y_i}{n}$$

$$b = \frac{Q_{xy}}{Q_{XX}}$$

$$a = \bar{y} - b\bar{x}$$

$$s_{y,x} = \sqrt{\frac{\sum_{i=1}^{n}\left[y_i - (bx_i + a)\right]^2}{n-2}}$$

$$s_{x,0} = \frac{s_{y,x}}{b}$$

$$y_k = a + s_{y,x} t_{f,a} \sqrt{\frac{1}{n} + \frac{1}{m} + \frac{\bar{x}^2}{Q_{xx}}}$$

$$LOD = \frac{y_k - b}{a}$$

$$LOQ = 3 \times s_{x,0} t_{f,a} \sqrt{\frac{1}{n} + \frac{1}{m} + \frac{(3 \times LOD - x)^2}{Q_{xx}}}$$
